# Chemical and structural changes associated with Cu-catalyzed alkaline-oxidative delignification of hybrid poplar

**DOI:** 10.1186/s13068-015-0300-5

**Published:** 2015-08-20

**Authors:** Zhenglun Li, Namita Bansal, Ali Azarpira, Aditya Bhalla, Charles H Chen, John Ralph, Eric L Hegg, David B Hodge

**Affiliations:** Department of Chemical Engineering and Materials Science, Michigan State University, East Lansing, MI USA; DOE-Great Lakes Bioenergy Research Center, Michigan State University, East Lansing, MI USA; Department of Biochemistry and Molecular Biology, Michigan State University, East Lansing, MI USA; DOE-Great Lakes Bioenergy Research Center, University of Wisconsin, Madison, WI USA; Department of Biochemistry, University of Wisconsin, Madison, WI USA; Department of Biosystems and Agricultural Engineering, Michigan State University, East Lansing, WI USA; Division of Sustainable Process Engineering, Luleå University of Technology, Luleå, Sweden; College of Agricultural Sciences, Oregon State University, Corvallis, OR USA; Department of Materials Science and Engineering, Johns Hopkins University, Baltimore, MD USA

**Keywords:** Plant cell walls, Pretreatment, Catalytic oxidation, Lignin, Alkaline hydrogen peroxide (AHP) pretreatment, NMR spectroscopy, Electron microscopy

## Abstract

**Background:**

Alkaline hydrogen peroxide pretreatment catalyzed by Cu(II) 2,2′-bipyridine complexes has previously been determined to substantially improve the enzymatic hydrolysis of woody plants including hybrid poplar as a consequence of moderate delignification. In the present work, cell wall morphological and lignin structural changes were characterized for this pretreatment approach to gain insights into pretreatment outcomes and, specifically, to identify the extent and nature of lignin modification.

**Results:**

Through TEM imaging, this catalytic oxidation process was shown to disrupt cell wall layers in hybrid poplar. Cu-containing nanoparticles, primarily in the Cu(I) oxidation state, co-localized with the disrupted regions, providing indirect evidence of catalytic activity whereby soluble Cu(II) complexes are reduced and precipitated during pretreatment. The concentration of alkali-soluble polymeric and oligomeric lignin was substantially higher for the Cu-catalyzed oxidative pretreatment. This alkali-soluble lignin content increased with time during the catalytic oxidation process, although the molecular weight distributions were unaltered. Yields of aromatic monomers (including phenolic acids and aldehydes) were found to be less than 0.2 % (wt/wt) on lignin. Oxidation of the benzylic alcohol in the lignin side-chain was evident in NMR spectra of the solubilized lignin, whereas minimal changes were observed for the pretreatment-insoluble lignin.

**Conclusions:**

These results provide indirect evidence for catalytic activity within the cell wall. The low yields of lignin-derived aromatic monomers, together with the detailed characterization of the pretreatment-soluble and pretreatment-insoluble lignins, indicate that the majority of both lignin pools remained relatively unmodified. As such, the lignins resulting from this process retain features closely resembling native lignins and may, therefore, be amenable to subsequent valorization.

**Electronic supplementary material:**

The online version of this article (doi:10.1186/s13068-015-0300-5) contains supplementary material, which is available to authorized users.

## Background

Non-renewable fossil resources have fueled economic development for the past two centuries and are a significant contributor to high standards of living around the world [1]. To meet future needs for the growing global demand for fuels, chemicals, polymers, and materials, new renewable resources must be accessed and new technologies employed in a sustainable fashion. Plant biomass accumulates and stores both solar energy and organic carbon through photosynthetic carbon fixation. As such, plant biomass results from efficient natural pathways for harnessing solar energy and can be utilized as a renewable source for reduced carbon for the production of biobased fuels and products to augment products that are currently derived from fossil resources [2].

Plant biomass is a highly heterogeneous material composed of multiple cell wall biopolymers that are associated primarily by non-covalent interactions and with composition and properties varying by cell and tissue type [3]. A diverse range of approaches is available for the conversion of plant cell wall-derived renewable fuels and chemicals. Many of these utilize a cell wall deconstruction strategy involving chemical pretreatment followed by enzymatic hydrolysis of the cell wall polysaccharides into sugar intermediates that can be subsequently utilized in chemical or biological conversion processes [4–7]. Structural and transport tissues comprise the bulk of the mass of hardwoods such as hybrid poplar, and are responsible, unless subjected to a cell wall-modifying pretreatment, for the substantial resistance to hydrolysis by cellulolytic enzymes [8]. Pretreatments improve hydrolysis through a combination of chemical and morphological changes to the cell wall that may include lignin and hemicellulose solubilization and/or relocalization that provides improved accessibility of cell wall polysaccharides to hydrolytic enzymes [9]. The higher order structure of the cell wall strongly impacts its response to deconstruction and conversion, and cell wall heterogeneity within woody plants has been implicated in differing responses to deconstruction [10]. The cell walls of woody plants often exhibit higher recalcitrance to these pretreatment processes than graminaceous plants due to higher lignin contents and denser vascular structures [11]. Acid sulfite pulping [12–14], alkaline pulping [15], organosolv pulping [16], dilute sulfuric acid pretreatment [17], liquid hot water pretreatment [18, 19], and steam pretreatment [20] have all been successfully employed as pretreatments for woody biomass, although these processes typically require higher chemical loadings, higher temperatures, and/or longer treatment times to achieve comparable hydrolysis yields to those from grasses. Chemical pulping technologies may require substantially less severe reaction conditions when adapted as pretreatments because high levels of delignification are not required [15].

Alkaline-oxidative treatments are ideally suited for the delignification of feedstocks such as woody biomass. These processes have been utilized commercially for wood pulp delignification and bleaching since the early 1980s [21], and for the commercial production of vanillin derived from the lignin removed during sulfite pulping of softwoods since the 1940s [22]. In addition, homogeneous catalysts and activators of O_2_ and H_2_O_2_ have been extensively investigated for their abilities to enhance delignification, for pulp brightening, and for their selectivities toward the desired lignin-directed oxidation reactions versus cellulose scission reactions [23–29]. In one study, Korpi et al. screened 189 different metal–ligand combinations for the oxidation of veratryl alcohol to veratraldehyde and found that copper complexes of 2,2′-bipyridine and 1,10-phenanthroline were especially active [30, 31]. These complexes were also reported to be effective delignification catalysts when H_2_O_2_ [32], O_2_ [33, 34], or both [35] were used as the oxidant. All approaches exhibited concurrent cellulose depolymerization that, although detrimental in instances where the cellulose is to be utilized in materials applications in which fiber strength is a crucial property, may prove beneficial if the desired product is monomeric sugars.

Previously we demonstrated that alkaline hydrogen peroxide (AHP) pretreatment in the presence of complexes of copper(II) 2,2′-bipyridine (Cu-catalyzed AHP pretreatment) is an effective method for improving the enzymatic digestibility of switchgrass, silver birch, and hybrid poplar [36, 37]. This improvement is accompanied by a decreased content of Klason lignin in pretreated biomass [36] that is associated with better enzyme accessibility to polysaccharides [38]. Similar associations have been reported for NaOH-only pretreatment [8], uncatalyzed AHP pretreatment [39], alkaline-oxidative delignification [40], and catalytic oxidative pretreatment with molecular oxygen using CuSO_4_ and 1,10-phenanthroline [34]. Despite the demonstrated efficacy of Cu-catalyzed AHP pretreatment in increasing lignin removal and enzymatic hydrolysis yields, the details of the structural and chemical modifications to the cell wall presumably responsible for these improvements remain unclear. To obtain a better understanding of the effects of Cu-catalyzed AHP pretreatment on both the cell walls and the pretreatment-solubilized compounds, a series of characterization techniques were employed. These included transmission electron microscopy (TEM) to investigate the structural changes in the plant cell wall and, as it turned out, to reveal the appearance of nanoscale particles within cell walls. This imaging was coupled with energy-dispersive X-ray spectroscopy (EDS) and electron energy-loss spectroscopy (EELS) to identify the elemental composition of these particles and verify the presence of Cu. NMR was used to structurally characterize the lignin fractions solubilized by the pretreatment as well as the residual insoluble lignin. LC–MS was employed to identify and quantify solubilized lignin-derived aromatics generated during pretreatment. Together, these data were integrated to provide insights into the Cu-catalyzed AHP process as well as the structural and chemical changes in the plant cell wall polymers that enhance enzymatic digestibility.

## Results and discussion

### Modifications to cell wall ultrastructure

Although uncatalyzed AHP has been shown to be quite effective at delignification, and substantially improves enzymatic hydrolysis yields of monomeric sugars for corn stover, it has minimal effect on hardwoods [41]. For example, uncatalyzed AHP pretreatment of hybrid poplar, using H_2_O_2_ loadings in the range of 125–500 mg/g biomass, results in only 10–36 % lignin removal and 18–30 % hydrolysis yields [36, 39]. The addition of copper(II) 2,2′-bipyridine [Cu(bpy)] complexes during AHP pretreatment, however, significantly increases both lignin removal (by up to 40 %) and glucose hydrolysis yields (by more than 60 %), even with short pretreatment times and low H_2_O_2_ loadings [37]. This removal of lignin has obvious structural implications for the accessibility of hydrolytic enzymes to cell wall polysaccharides.

**Fig. 1:**
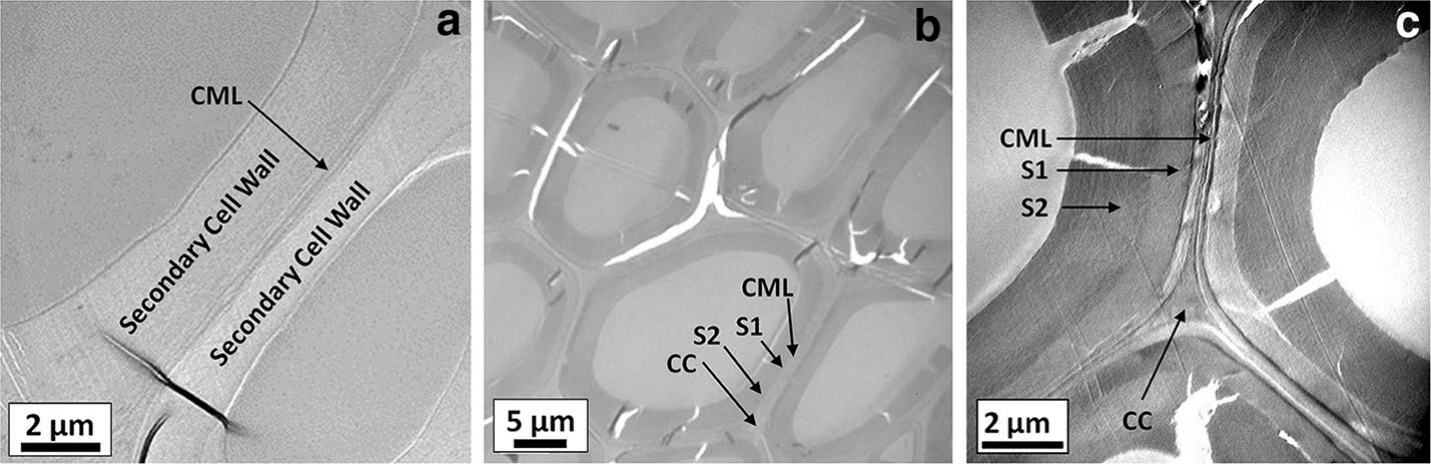
TEM micrographs of cross sections of **a** untreated and **b**, **c** AHP-only pretreated hybrid poplar cell wall. The micrographs in **b** and **c** exhibit some dislocations between and within cell walls as a consequence of AHP pretreatment.

Recently, a number of approaches have been applied to relate cell wall supramolecular organization to its recalcitrance and to determine how cell wall structure is altered by pretreatment, as reviewed [42]. Notably, TEM imaging has been used to characterize corn stover cell wall structural changes associated with pretreatments by dilute acid [43], and anhydrous ammonia [44], as well as acid chlorite delignification [45]. TEM micrographs of untreated hybrid poplar (Fig. 1a) and AHP-only pretreated hybrid poplar (Fig. 1b, c) showed identifiable features of the cell walls comprising wall layers that include secondary cell wall layers (S1 and S2) and the compound middle lamella (CML) as well as cell corners (CC) and individual lumens. The dark black stripes observed in the micrographs are artifacts introduced during ultramicrotome sectioning and KMnO_4_ staining [46]. Following uncatalyzed AHP pretreatment (Fig. 1b, c), the cell walls retained much of their structural integrity, as seen by their similarities to those of untreated cell walls. The only notable changes were that dislocations formed between the middle lamellae and the primary cell walls, possibly due to removal of some lignin and pectic polysaccharides during pretreatment [36, 39].

**Fig. 2:**
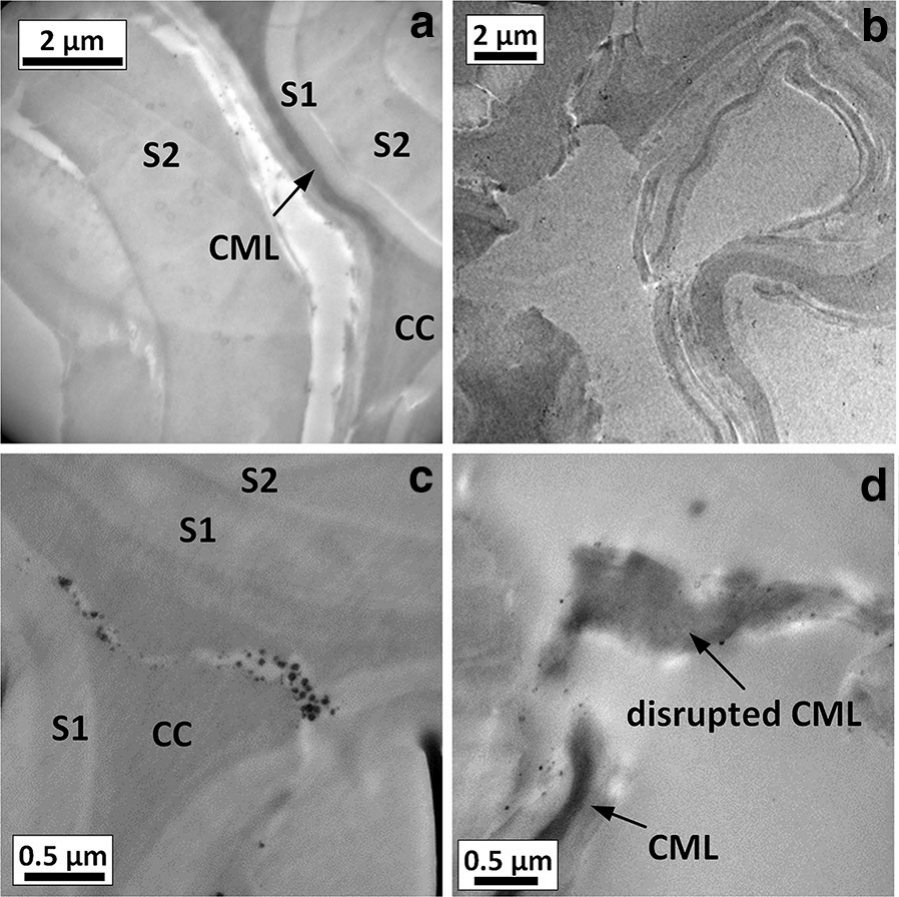
TEM characterization of hybrid poplar cell wall after Cu-catalyzed AHP pretreatment demonstrating **a** delamination and **b** dislocations of cell wall layers along with accumulation of nanoparticles in disrupted regions (**c**, **d**).

Addition of Cu(bpy) complexes during AHP pretreatment of hybrid poplar improved delignification and enzymatic hydrolysis yields [36]. TEM imaging showed that significant cell wall structural changes had occurred (Fig. 2). One obvious change was the major dislocations in the cell wall, examples of which are shown in Fig. 2a, and the formation of fractures in which the secondary cell wall layers were perturbed. Fractures and disruptions were also observed in other lignin-rich regions including cell corners and compound middle lamellae, suggesting that the structural changes may be caused by lignin modification and/or removal. Plant cell wall imaging following dilute acid [43] and anhydrous ammonia [44] pretreatments of delignified wood pulps [47], and following acid chlorite for delignified corn stover [45], has shown both dislocations and delaminations within the secondary cell wall.

Lignin removal from the cell wall can be achieved through relocalization by a combination of solvent and temperature [44, 48], or through low- [45] or high-temperature delignification [49]. An important consideration for Cu-catalyzed AHP pretreatment is that it was performed at 25–30 °C, temperatures well below the lignin glass transition (100–170 °C) [50]. As a consequence, the lignin removal we observed is primarily due to chemical modification and/or solvent effects rather than thermal effects. We also observed that electron-opaque particles with diameters in the range of 20–100 nm were often co-localized with the modified regions of cell walls (Fig. 2c, d). These particles were not found in untreated hybrid poplar or AHP-pretreated poplar (Fig. 1). As such, they can be hypothesized to originate from the catalyst rather than as artifacts introduced during the TEM sample preparation. This suggests that copper is involved in the observed cell wall modification (e.g., via lignin oxidation).

### Elemental profiling of pretreated cell walls

**Fig. 3:**
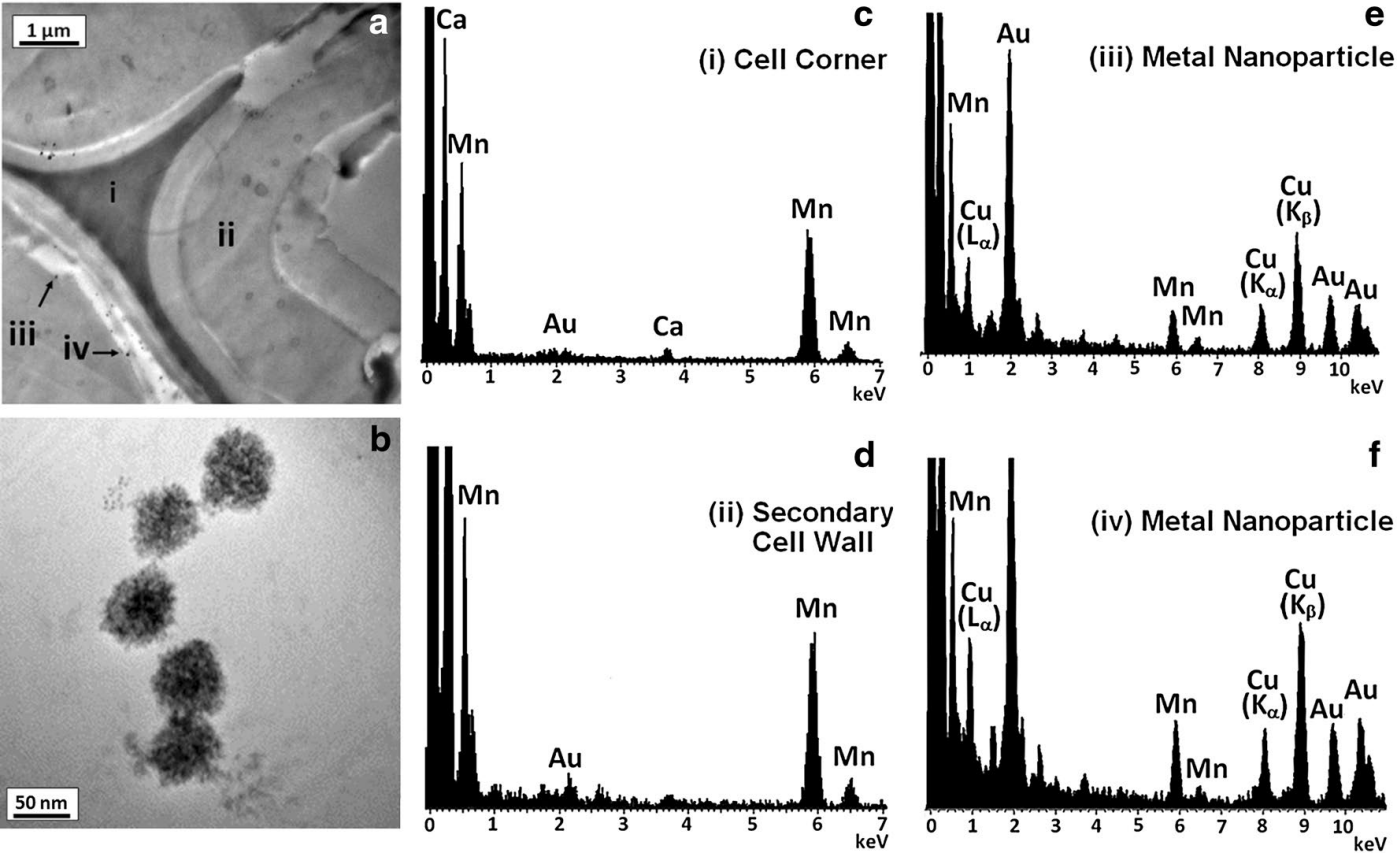
TEM micrographs of hybrid poplar cell wall (**a**) and high-resolution image of an electron-opaque aggregate (**b**) together with acquired X-ray EDS spectra of select regions within this sample (**c**–**f**).

When combined with electron microscopy, in situ elemental profiling using energy-dispersive X-ray spectroscopy (EDS) and electron energy-loss spectroscopy (EELS) can provide chemical characterization at a spatial resolution on the order of 100 nm [51]. To characterize the elemental composition of the nano-scale particles observed in the TEM images, EDS spectra were acquired at different locations in a TEM sample (Fig. 3a), including at a cell corner (identified as area i in Fig. 3a), within a secondary cell wall (area ii), and over the previously described particles (areas iii, iv). TEM images at high magnification show that these particles are aggregates with dendritic structures and diameters on the order of 50 nm (Fig. 3b). A comparison of the EDS spectra reveals both similarities and differences in elemental composition of the cell wall regions (Fig. 3c–f). Mn peaks are apparent in all four spectra and are a consequence of the permanganate staining, whereas the Au peaks correspond to the X-ray emissions from the grid that supports the TEM sample. The EDS spectrum from the cell corner (area i) has a strong Ca L-edge peak indicating the presence of Ca ions that are known to complex with pectin [52]. Ca K-edge peaks (3.7 keV) are also present at a lower relative abundance in the spectra of the other cell areas. For areas ii and iii where electron-opaque particles were analyzed, the EDS spectra feature characteristic peaks for Cu. The Cu L-edge and K-edge peaks are not seen in the EDS spectra of either the cell corner (area i) or the secondary cell wall (area ii). EELS was used to identify the oxidation state of the Cu-containing particles (Additional file 1: Figure S1), which shows the spectrum of a Cu-containing particle with the pre-edge background subtracted. The white-line intensity (i.e., the sharp threshold peaks) of the Cu L_2,3_ edge indicates that the majority of the Cu is in the Cu(I) oxidation state, while the relatively low white-line intensity of L_3_ implies that Cu(0) is also present.

The identification of Cu-containing particles suggests that the Cu catalyst is localized in the cell wall matrix at sites corresponding to those with significant structural modification. One compelling interpretation of this result is that the soluble Cu catalysts diffuse into the porous cell wall matrix during pretreatment and subsequently catalyze the formation of localized reactive oxygen species that may be involved in the oxidative delignification and structural modification of the cell wall in their vicinity. Although the active catalytic complexes have not yet been identified, one possibility is that the oxidation reactions are accelerated by Cu complexes that catalyze the decomposition of H_2_O_2_ [53] and/or activate H_2_O_2_ via the formation of Cu-peroxide complexes [54].

Whether the formation of the Cu-containing particles occurs during pretreatment or sample preparation is still unknown. A number of scenarios can explain the results. The solubility and speciation of the Cu(bpy) complexes are a function of pH, concentration, and ligand to metal ratio [55], with Cu(bpy) complexes being substantially more soluble at the alkaline pH where pretreatment occurs. As a consequence, Cu-containing particles may precipitate at the neutral pH where sample fixation is performed. Another possibility is that the observed Cu-containing particles provide indirect evidence of catalytic activity as the soluble Cu(bpy) complexes are reduced from Cu(II) to the observed Cu(I) and Cu(0) oxidation states during the pretreatment process and are subsequently deposited as insoluble aggregate particles. Whether these particles are catalytically active or inactive remains to be determined. Furthermore, the reduction of Cu(II) by the incident electrons during TEM imaging cannot be ruled out.

### Characterization of solubilized cell wall biopolymers and phenolic monomers

**Fig. 4:**
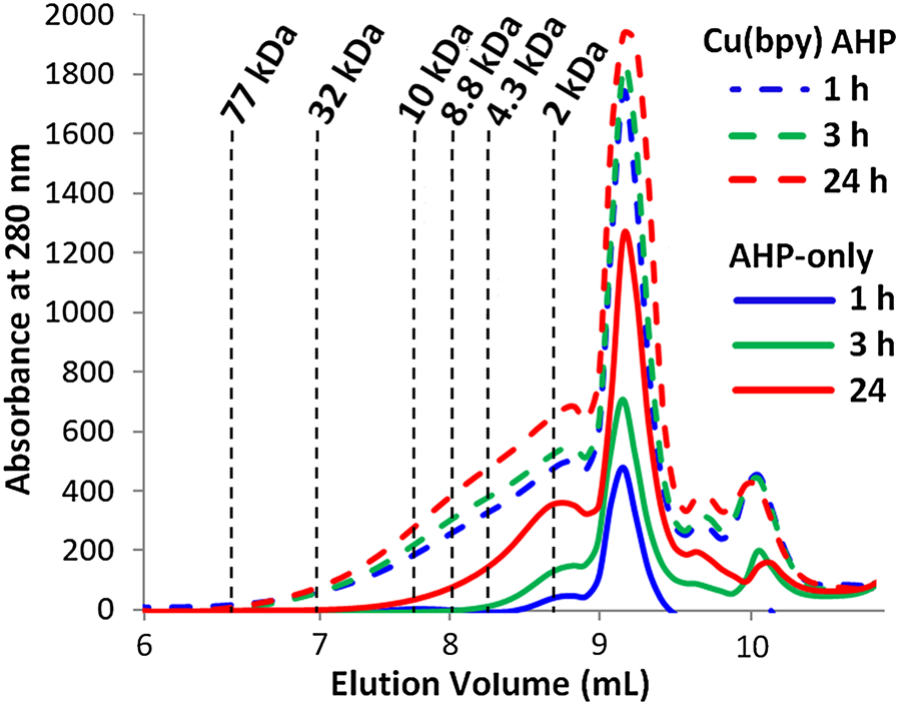
SEC elution profiles for plant cell wall polymers solubilized during pretreatments referenced to elution times for polystyrene standards. For reference, the phenolic monomers vanillin, vanillic acid, syringaldehyde, syringic acid, and *p*-hydroxybenzoic acid elute at elution volumes between 9.1 and 9.35 mL.

Our previous work demonstrated that a significant fraction of the lignin can be removed from the cell wall during Cu-catalyzed AHP pretreatment [36]. This delignification could result from depolymerization (which would increase the phenolic hydroxyl content and the solubility of the lignin in alkali) [56], oxidation of the lignin (which in many cases increases the hydrophilicity of the lignin), or a combination of the two processes. Cell wall biopolymer structural changes associated with this pretreatment were assessed using multiple analytical approaches. In the first approach, the relative abundance and molecular weight distributions of the lignin solubilized during pretreatment were determined by size-exclusion chromatography (SEC) as a function of pretreatment time (Fig. 4). A single large peak representing phenolic monomers and oligomers eluted below an apparent molecular weight of 1,000 Da. The clear trend from these elution profiles is that Cu-catalyzed AHP releases significantly more soluble lignin fragments than uncatalyzed AHP pretreatment, supporting our previous findings that lignin solubilization during the pretreatment results in increased enzymatic hydrolysis yields [37]. It should be noted, however, that carbonyl functional groups on oxidized lignins may also strongly absorb at 280 nm and result in differing response factors for lignins with differing levels of oxidation. Another key observation is that the molecular weight distributions of the solubilized lignins are not noticeably altered for either of the pretreatments over time (Fig. 4), indicating that soluble lignins are neither undergoing substantial depolymerization, nor repolymerization through condensation reactions.

**Fig. 5:**
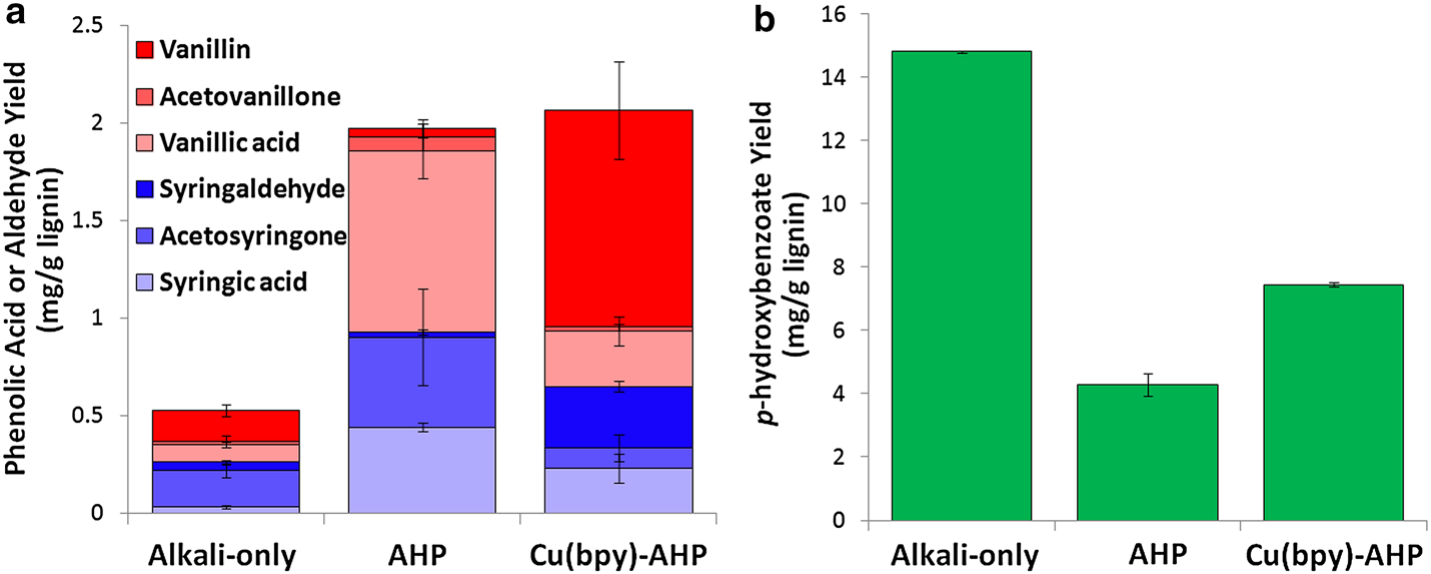
Yields of **a** phenolic acids and aldehydes (excluding *p*-hydroxybenzoate) and **b** *p*-hydroxybenzoate following alkali-only, AHP, and Cu-catalyzed AHP pretreatment.

The distribution and abundance of the phenolic monomers solubilized following pretreatment were quantitated by LC–MS and HPLC. These monomers arise primarily through the cleavage of ether bonds or by saponification of phenolic acids that acylate the lignin polymer. The distribution of phenolic monomers was found to be substantially different when the Cu catalyst was present, with aldehyde products favored over acids (Fig. 5a). This quantitative difference between the release of phenolic acids and aldehydes suggests that the Cu-catalyzed reaction may utilize a different reaction mechanism that results in less oxidation of the lignin polymer, yet yields more intra-lignin bond cleavage and lignin solubilization than uncatalyzed AHP. Another important result is that phenolic monomer yields reach only 2 mg/g lignin (i.e., mass of monomer to mass of cell wall lignin) for both AHP-only and Cu-catalyzed AHP pretreatment, and only 0.5 mg/g lignin by alkali-only pretreatment (Fig. 5a). In contrast, the complete scission of β–O–4-bonds is expected to generate maximum phenolic monomer yields ranging up to 50 % or more depending on properties such as the syringyl/guaiacyl (S/G) ratio and whether the lignins have undergone extensive condensation during processing or extraction [41, 57–60]. Thus, our results suggest that the cleavage of β–O–4-bonds is far from complete.

Plants within the family *Salicaceae*, including the genus *Populus*, are known to have lignins with *p*-hydroxybenzoate groups acylating the γ-OH of syringyl subunits [61, 62]. As expected, the most abundant phenolic monomer in all of the pretreatment liquors was *p*-hydroxybenzoate (Fig. 5b), as these esters are easily saponified during alkaline pretreatments. The results show that alkali-only treatment results in the highest yields (14.8 mg/g lignin) with AHP-only and Cu(bpy)-AHP releasing roughly one-third or one half of this quantity. The lower yields following the oxidative treatments are presumably due to the lower pHs during these treatments (due to the acidic contribution of H_2_O_2_) that may result in incomplete saponification of the *p*-hydroxybenzoate, although oxidative degradation/modification may also contribute to this difference.

### 2D HSQC NMR of whole cell walls and solubilized lignins

**Fig. 6:**
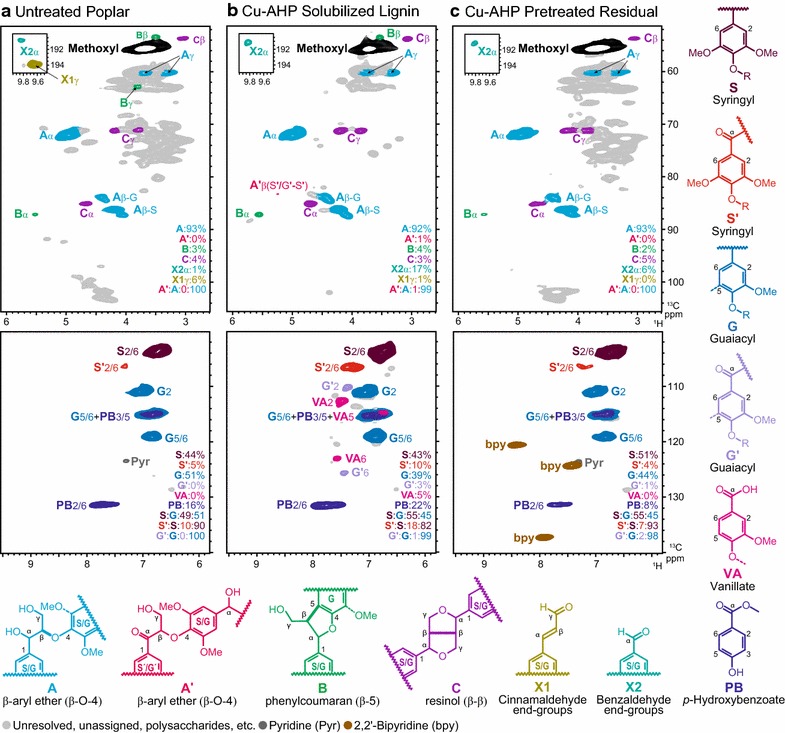
Partial 2D HSQC NMR spectra of **a** whole cell wall untreated poplar, **b** solubilized lignin, and **c** residual poplar cell walls following Cu-catalyzed AHP pretreatment. Contours are *colored* to match the structures for aromatic components.

We used 2D HSQC (heteronuclear single-quantum coherence) NMR spectroscopy to analyze lignin after Cu-catalyzed AHP pretreatment. NMR characterization was performed on untreated whole cell wall material from hybrid poplar (Fig. 6a), the lignin that was solubilized following 1 h of catalytic pretreatment and recovered via acid precipitation (Fig. 6b), and the residual insoluble cell wall material following pretreatment (Fig. 6c). Several important insights can be gained from these experiments. First, the NMR data provide evidence for lignin oxidation. The aromatic region of the pretreatment-solubilized lignin (Fig. 6b, lower panel) revealed a substantial increase in oxidized S and G units to their benzylic ketone analogues S′ and G′ plus new vanillate units (VA). The aliphatic region (Fig. 6b, upper panel) further supports these chemical changes. Although most of the correlations corresponding to β-ether units in the aliphatic region remained intact, correlations for the corresponding oxidized analogues A′ provide further evidence of benzylic oxidation. Whether this oxidation occurred prior to or following lignin solubilization cannot be established. Currently, we do not know how other lignin structures such as β–5-linked units (phenylcoumaran), and β–β-linked (resinol) units (structures B and C in Fig. 6) react, but such structures obviously remain intact in this fraction. We also noted that cinnamaldehyde end groups (X1) are completely absent from the oxidized lignin samples, whereas benzaldehyde end groups (X2) remain. Monomeric and oligomeric fragments with aryl-aldehyde and aryl-acid structures have been identified in milled wood lignins following catalytic oxidation [60], and also in lignosulfonates [63, 64]. Although the aromatic ring is inactivated toward oxidation due to carbonyl conjugation [65], the aryl α-carbonyl is susceptible to nucleophilic attack by hydroxyl groups followed by cleavage of the side-chain C_α_–C_β_ bond [60, 66]. Such cleavage will decrease the molecular weight of polymeric lignin and create hydrophilic lignin fragments with benzoate and benzaldehyde end-groups, consistent with the MS data (Fig. 5a). A second important insight is that lignin depolymerization is not extensive for pretreatment by Cu-catalyzed AHP. Using peak integrations as described previously [67], it can be observed that the β–O–4, β–5 and β–β linkages are still present in both the solubilized lignin (Fig. 6b) and in the residual pretreatment-insoluble lignin (Fig. 6a) in approximately the same ratio as in the native lignin. Together with the low yields of aromatic monomers observed in LC–MS (Fig. 5a) and the SEC studies that reveal an increase in solubilized lignins without a noticeable shift in the molecular weight distributions (Fig. 4), these NMR results strongly support a pathway in which Cu-catalyzed AHP pretreatment solubilizes and removes a fraction of the cell wall-bound lignin with minimal depolymerization and minimal oxidation of the residual lignin.

The lignins from Cu-catalyzed AHP pretreatment may be a promising source of lignin for the production of value-added products if integrated into a biorefining process. Specifically, two lignin streams with distinct structural properties are generated comprising pretreatment-solubilized lignins and residual pretreatment-insoluble lignins. The solubilized lignins (Fig. 6b) showed minimal depolymerization, exhibited minor oxidative modification, and would contain soluble xylan oligosaccharides unless these are further hydrolyzed and converted. The residual, pretreatment-insoluble lignin (Fig. 6c) exhibited minimal modification as a consequence of the pretreatment with structures closely resembling native lignins (albeit requiring recovery of bipyridine). Furthermore, by controlling the pretreatment time and the oxidation stoichiometry, it might be possible to control the molecular weight and chemical properties of solubilized lignins, customizing them for the production of functional materials and fine chemicals with targeted properties.

## Conclusions

Our previous work demonstrated that Cu-catalyzed AHP pretreatment of hybrid poplar at moderate chemical loadings and under mild conditions substantially improves hydrolysis yields relative to uncatalyzed AHP pretreatment. One of the primary outcomes of pretreatment is the solubilization of a fraction (less than 30 % under these reaction conditions) [36] of the cell wall-bound lignin that results in improved accessibility of cell wall carbohydrates to hydrolytic enzymes. The current work provided a number of insights into the structural changes that occur to the cell wall and cell wall biopolymers following Cu-catalyzed AHP pretreatment of hybrid poplar. Specifically, we demonstrated that the catalyzed pretreatment resulted in disrupted cell walls manifested by dislocations between individual cell walls as well as delaminations within cell walls and that copper-containing nanoparticles co-localized with these zones of disruption. We hypothesize that sorption of catalyst into the cell wall during pretreatment results in oxidation, solubilization, and removal of lignin resulting in observable cell wall disruptions and enhanced susceptibility to enzymatic hydrolysis. Consistent with this hypothesis, both LC–MS and NMR characterization of the solubilized lignins and the residual material following Cu-catalyzed AHP pretreatment revealed the presence of oxidized lignin fragments. Specifically, a fraction of the hydroxyl groups at the α-carbon in β–O–4-units were oxidized to carbonyls, and end-groups characteristic of hydrolytic cleavage of oxidized lignin side-chains were created, suggesting that depolymerization results in lignin solubilization and removal during the pretreatment. Intriguingly, whereas the pretreatment-solubilized lignins exhibited a more than threefold increase in the oxidation of the benzylic alcohol relative to native lignin (with correlation peak integrals increasing from 5 to 18 %), the extent of lignin oxidation was limited in the pretreatment-insoluble lignin, which resembled native lignins. Formation of the Cu-containing nanoparticles with oxidation states of Cu(I) and Cu(0) lower may be attributed to reduction of soluble Cu(bpy) complexes during pretreatment, although we cannot rule out the possibility that these particles are formed during sample preparation. Additionally, relative to the lignins generated during other pretreatments and/or delignification processes that are performed at elevated temperatures and often result in substantial lignin modification, the mildly oxidized lignins generated in this work retain features closely resembling native lignins and, as such, may add more value to an integrated biorefining process.

## Methods

### Biomass, pretreatment, and hydrolysis

Hybrid poplar (*Populus nigra* var. *charkoviensis* × *caudina* cv. NE-19) was grown at the University of Wisconsin Arlington Agricultural Research Station. Prior to pretreatment, a mixture of heartwood and sapwood of the 18-year-old poplar was hammer-milled to pass a 5-mm screen. Procedures for the compositional analysis and the pretreatment of the biomass were previously reported [36]. For AHP pretreatment, hybrid poplar (0.5 g) was pretreated in 5 mL aqueous aliquots of 10.0 g H_2_O_2_/L (10 % wt/wt loading) and 10.8 g NaOH/L (final pH of approximately 11.7) at 30 °C for 1 h unless otherwise noted. During the pretreatment, the samples were agitated at 180 rpm in an orbital shaker. For Cu-catalyzed AHP pretreatment, 5 mM CuSO_4_ and 25 mM 2,2′-bipyridine prepared as described previously [36] were included in the 5 mL aliquot during pretreatment. For alkali-only pretreatment, 0.5 g of hybrid poplar was pretreated in 5 mL aqueous aliquots of 10.8 g NaOH/L.

### TEM imaging and elemental profiling of pretreated cell walls

Structural modification of hybrid poplar cell wall by pretreatment was studied using transmission electron microscope (TEM) combined with energy-dispersive X-ray spectroscopy (EDS) and electron energy-loss spectroscopy (EELS). The conditions used for pretreatment were identical to those used to prepare SEC samples. Cell wall samples of untreated hybrid poplar and hybrid poplar treated with AHP and Cu-catalyzed AHP for 24 h were air dried and fixed in 0.1 M pH 7.0 phosphate buffer [68] containing 2.5 % (w/w) glutaraldehyde and 2.5 % (w/w) paraformaldehyde. The fixed cell wall samples were embedded in Spurr epoxy resin (Poly/Bed 812, Polysciences) and sectioned to 100 nm thickness using a PowerTome XL ultramicrotome (Boeckeler Instruments, Tucson, AZ, USA). Thin sections were placed on 150 mesh gold grids with Formvar/carbon support film (Electron Microscopy Sciences, PA, USA) and stained in 1 % aqueous solution of KMnO_4_ for 60 s. Samples were then rinsed with deionized water to remove excess stain. Bright field TEM micrographs and EELS spectra were acquired under a JEOL 2200FS 200 kV field emission TEM (Peabody, MA, USA) fitted with a Gatan (Warrendale, PA, USA) digital multi-scan camera. EDS spectra were acquired using an Oxford INCA system (Oxford Instruments, Abington, UK) coupled with the TEM.

### Analysis or pretreatment liquors

For analysis by size exclusion chromatography (SEC) or LC–MS, pretreatment liquors at alkaline pH were filtered through a 0.22-µm mixed cellulose ester membrane filter (EMD Millipore, Billerica, MA, USA). SEC analysis was performed using an Agilent 1100 HPLC equipped with an Ultrahydrogel 250 column (Waters, Milford, MA, USA) as described previously [69] only using a mobile phase flowrate of 0.6 mL/min. Aqueous solutions of monodisperse sodium polystyrene sulfonate (Sigma-Aldrich, St. Louis, MO, USA) of known molar mass (2,000, 4,300, 6,800, 10,000, 32,000, and 77,000 Da) were used as calibration standards.

Samples for LC–MS analysis were prepared as described above for SEC analysis except that the concentrations of CuSO_4_ and 2,2′-bipyridine during pretreatment were 2 mM and 4 mM, respectively. For LC–MS analysis, 10 µL of undiluted pretreatment liquor sample was adjusted to pH 2.0 with formic acid and was injected into a XEVO G2SQTOF mass spectrometer in combination with a Waters Acquity UPLC system, and equipped with an ESI interface capable of operating in both positive- and negative-ion modes. Chromatographic separation was carried out on a Thermo BetaBasic 100 × 2.1 mm C18 column (Thermo Fisher Scientific, Waltham, MA, USA) maintained at 40 °C. The binary solvent gradient consisted of 0.1 % formic acid in water (solvent A) and 100 % methanol (solvent B) in the following gradient: 95 % solvent A for the first 3 min, 50 % solvent A over the next 1 min, 30 % solvent A over next 2 min, and 5 % solvent A over the final 2 min. The column was then returned to 95 % solvent A and equilibrated for 2 min prior to the next injection. A solvent delay of 2 min was used to prevent saturation of the detector with the sample solvent. The negative-ion mode mass spectrometry conditions were constant during all experiments with a voltage of −2.25 kV and a desolvation temperature of 350 °C. MassLynx software (Waters) version 4.1 was used for system control and data acquisition. The raw data acquired were processed using the TargetLynx application. Pure standards for vanillin, vanillic acid, acetovanillone, syringaldehyde, syringic acid, acetosyringone, and *p*-hydroxybenzoate (Sigma-Aldrich, St. Louis, MO, USA) were used to validate peak compound identification and for quantitation.

For quantitation of *p*-hydroxybenzoate, samples were prepared following the same procedure as that for the LC–MS analysis, but the samples were then analyzed via high-performance liquid chromatography (Agilent 1260 LC equipped with an Agilent Poroshell 120 EC-C18 column (4.6 × 50 mm) and a diode array detector (DAD). Integration of the *p*-hydroxybenzoate peak at 280 nm and comparison to a standard curve was used for quantitation. A binary isocratic solvent system was utilized consisting of 80:20 solvent C to solvent D, where solvent C is acetonitrile with 0.1 % water, and solvent D is acetonitrile with 0.1 % trifluoroacetic acid.

### NMR analysis of whole cell walls and pretreatment-solubilized lignin

Following the pretreatment, the aqueous phase was separated from the solid phase (i.e., the insoluble portion of pretreated poplar) via filtration and the filtrate was acidified to pH 2.0 with 72 % (w/w) sulfuric acid. The precipitate from the acidified filtrate was recovered via centrifugation and washed with a large volume of aqueous sulfuric acid (pH 2.0) followed by a final washing step of resuspending and decanting the lignin sample in pH-neutral deionized water. The washed lignin precipitate was lyophilized prior to NMR analyses. The 2D HSQC NMR spectra of three types of samples (untreated hybrid poplar, recovered solubilized lignins and the insoluble portion of pretreated poplar) were acquired and analyzed as previously described by Kim et al. [70].

Untreated and pretreated samples were prepared for gel-state NMR as previously described [70]. In brief, the dried sample was pre-ground for 1 min in a Retsch MM400 mixer mill at 30 Hz, using zirconium dioxide (ZrO_2_) vessels (10 mL) containing ZrO_2_ ball bearings (2 × 10 mm). The ground material was extracted with distilled water (1 h, 3 times) and 80 % of ethanol (1 h, 3 times) with ultrasonication. The cell walls were dried and finely ball-milled using a PULVERISETTE 7 (Fritsch, Idar-Oberstein, Germany) mill at 600 rpm with ZrO_2_ vessels (50 mL) containing with ZrO_2_ ball bearings (10 × 10 mm). Each sample (200 mg) was milled for 1 h 40 min in 10 min intervals with 5 min interval breaks. The ball-milled samples (50 mg of each) were transferred into 5 mm NMR tubes and gels formed using DMSO-*d*_*6*_/pyridine-*d*_*5*_ (4:1, v/v, 0.5 mL) with sonication (30 min).

NMR spectra were acquired on a Bruker BioSpin (Billerica, MA, USA) AVANCE 700 MHz spectrometer equipped with a cryogenically cooled 5 mm triple-resonance ^1^H/^13^C/^15^N TXI gradient probe with inverse geometry (^1^H coils closest to the sample). The central DMSO solvent peak was used as internal reference (δ_C_ 39.5, δ_H_ 2.49 ppm). The ^1^H–^13^C correlation experiment was an adiabatic HSQC experiment (Bruker standard pulse sequence ‘hsqcetgpsisp.2'; phase-sensitive gradient-edited 2D HSQC using adiabatic pulses for inversion and refocusing) [71]. HSQC experiments were carried out using the following parameters: acquired from 9 to 1 ppm in F2 (^1^H) with 1,200 data points (acquisition time 200 ms), 160–10 ppm in F1 (^13^C) with 512 increments (F1 acquisition time 13.6 ms) of 32 scans with a 1-s interscan delay; the d_24_ delay was set to 0.86 ms (1/8 J, J = 145 Hz). Volume integration of contours in HSQC plots used Bruker’s TopSpin 3.1 (Mac) software. Assignments of peaks from NMR spectra were based on previous publications [70, 72].

## Additional file

Additional file 1:
**Figure S1.** EELS spectrum of the Cu-containing nanoparticles showing the Cu L2,3 edge providing evidence that the Cu in these particles is primarily in the Cu(I) oxidation state with contributions by Cu(0). **Figure S2.** Partial 2D HSQC NMR spectra of (a) whole cell wall untreated poplar, (b) solubilized lignin, and (b) residual poplar cell walls following Cu-catalyzed AHP pretreatment. Contours are colored to match the structures for aromatic components. [This is the same as Fig. 6 in the main paper except that important polysaccharide correlations have been assigned].

## Abbreviations

AHP: alkaline hydrogen peroxide
bpy: 2,2′-bipyridine
DAD: diode array detector
EDS: energy-dispersive X-ray spectroscopy
EELS: electron energy loss spectroscopy
ESI: electrospray ionization
HPLC: high-performance liquid chromatography
HSQC: heteronuclear single-quantum coherence
LC–MS: liquid chromatography–mass spectrometry
NMR: nuclear magnetic resonance
SEC: size exclusion chromatography
TEM: transmission electron microscopy
UPLC: ultra performance liquid chromatography
