# Supplementary material for: Chemical and structural changes associated with Cu-catalyzed alkaline-oxidative delignification of hybrid poplar
Source: Biotechnol Biofuels. 2015 Aug 20;8:123. doi: 10.1186/s13068-015-0300-5 (PMC4546027; doi:10.1186/s13068-015-0300-5)
Supplement: Additional file 1: — Figure S1. EELS spectrum of the Cu-containing nanoparticles showing the Cu L2,3 edge providing evidence that the Cu in these particles is primarily in the Cu(I) oxidation state with contributions by Cu(0). Figure S2. Partial 2D HSQC NMR spectra of (a) whole cell wall untreated poplar, (b) solubilized lignin, and (b) residual poplar cell walls following Cu-catalyzed AHP pretreatment. Contours are colored to match the structures for aromatic components. [This is the same as Fig. 6 in the main paper except that important polysaccharide correlations have been assigned]. [file 13068_2015_300_MOESM1_ESM.pdf]

ADDITIONAL FILES

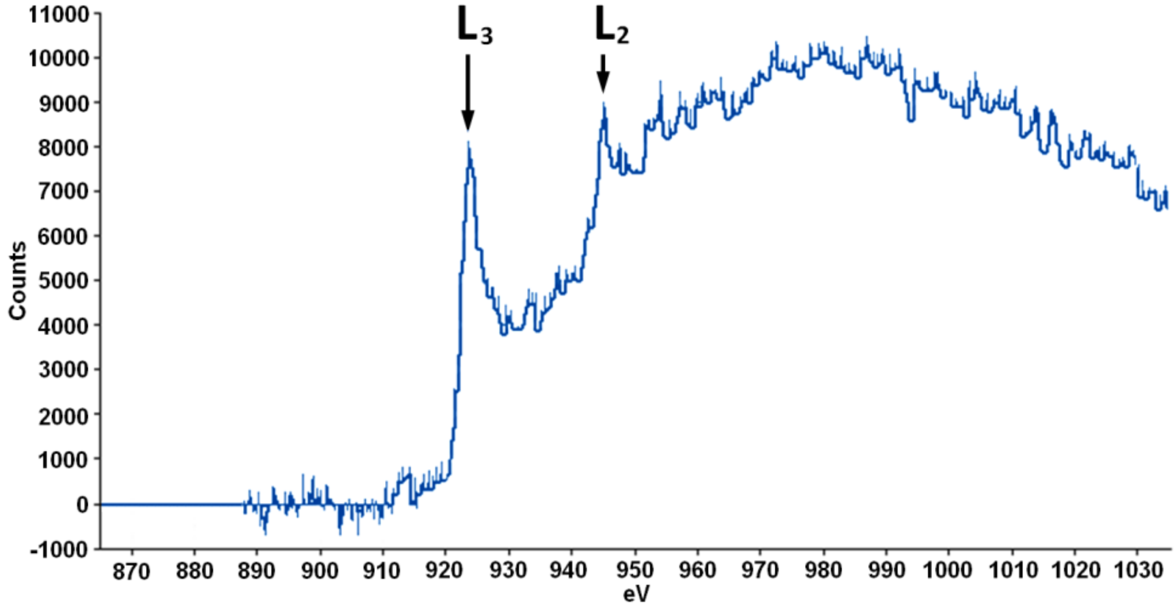

Supplemental Figure S1. EELS spectrum of the Cu-containing nanoparticles showing the Cu $L_{2,3}$ edge providing evidence that the Cu in these particles is primarily in the Cu(I) oxidation state with contributions by Cu(0).

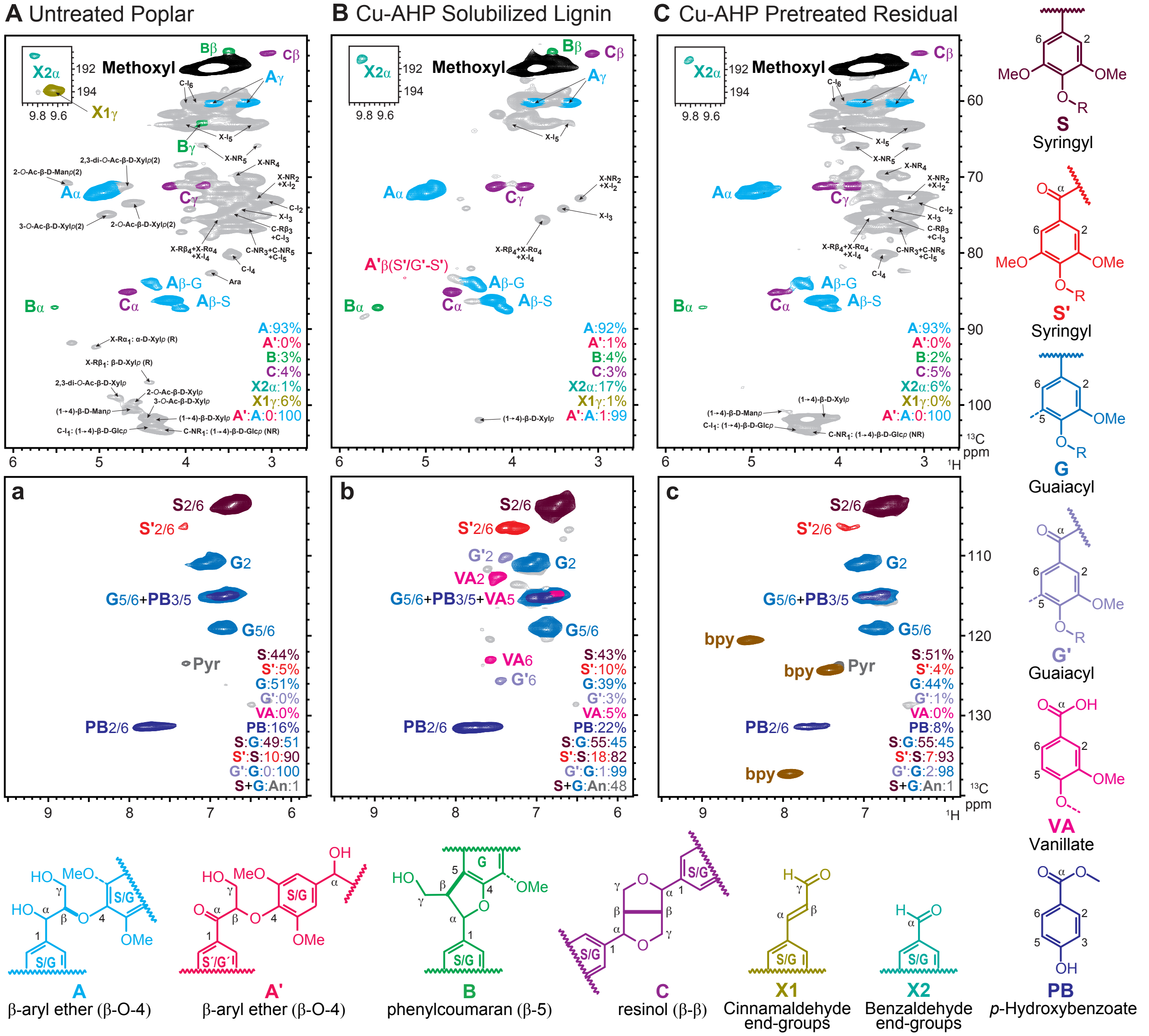

Unresolved, unassigned, polysaccharides, etc.
Pyridine (Pyr)
2,2'-Bipyridine (bpy)

*C-I, cellulose internal unit; C-NR, cellulose non-reducing end unit; C-Rα, cellulose α reducing end unit; C-Rβ, cellulose β reducing end unit; X-I, xylose internal unit; X-NR, xylan non-reducing end unit; X-Rα, xylan α reducing end unit; X-Rβ, xylan β reducing end unit; R, reducing end; NR, non- reducing end (Kim et al, RSC Adv., 2014, 4, 7549–7560).

Supplemental Figure S2. Partial 2D HSQC NMR spectra of (a) whole cell wall untreated poplar, (b) solubilized lignin, and (b) residual poplar cell walls following Cu-catalyzed AHP pretreatment. Contours are colored to match the structures for aromatic components. [This is the same as Figure 6 in the main paper except that important polysaccharide correlations have been assigned.]
